# Comparison of Frailty and Chronological Age as Determinants of the Murine Gut Microbiota in an Alzheimer’s Disease Mouse Model

**DOI:** 10.3390/microorganisms11122856

**Published:** 2023-11-24

**Authors:** Laura Malina Kapphan, Vu Thu Thuy Nguyen, Isabel Heinrich, Oliver Tüscher, Pamela Passauer, Andreas Schwiertz, Kristina Endres

**Affiliations:** 1 Department of Psychiatry and Psychotherapy, University Medical Center Johannes Gutenberg-University Mainz, 55131 Mainz, Germany; lauramalina@aol.com (L.M.K.); vuthuthuy.nguyen@outlook.de (V.T.T.N.); isabel.heinrich@unimedizin-mainz.de (I.H.); oliver.tuescher@unimedizin-mainz.de (O.T.); 2 MVZ Institut für Mikroökologie GmbH, 35745 Herborn, Germany; pamela.passauer@mikrooek.de (P.P.); andreas.schwiertz@mikrooek.de (A.S.)

**Keywords:** ageing, Alzheimer’s disease, *Bacteroides*, frailty, microbiota

## Abstract

The ageing of an organism is associated with certain features of functional decline that can be assessed at the cellular level (e.g., reduced telomere length, loss of proteostasis, etc.), but also at the organismic level. Frailty is an independent syndrome that involves increased multidimensional age-related deficits, heightens vulnerability to stressors, and involves physical deficits in mainly the locomotor/muscular capacity, but also in physical appearance and cognition. For sporadic Alzheimer’s disease, age per se is one of the most relevant risk factors, but frailty has also been associated with this disease. Therefore, we aimed to answer the two following questions within a cross-sectional study: (1) do Alzheimer’s model mice show increased frailty, and (2) what changes of the microbiota occur concerning chronological age or frailty? Indeed, aged 5xFAD mice showed increased frailty compared to wild type littermates. In addition, 5xFAD mice had significantly lower quantities of *Bacteroides* spp. when only considering frailty, and lower levels of *Bacteroidetes* in terms of both frailty and chronological age compared to their wild type littermates. Thus, the quality of ageing—as assessed by frailty measures—should be taken into account to unravel potential changes in the gut microbial community in Alzheimer’s disease.

## 1. Introduction

Ageing is a phenomenon that affects all organisms. However, the quality of ageing can vary widely within a species, ranging from being fit until death (‘well-ageing’) to being frail and morbid. Age is one of the most important risk factors for the dominant, sporadic form of Alzheimer’s disease (AD) [1]. Frailty itself, the functional decline observed in ageing, is also a well-established risk factor for cognitive decline and dementia in humans [2,3,4,5]. A longitudinal study reported that 10-year trajectories of frailty development were steeper in patients with dementia and mild cognitive impairment (MCI) than in healthy controls. This finding persisted even after controlling for relevant covariates, including the neuropathological index [6]. Similarly, signs of increased frailty can be found in AD mouse models. In APP23 mice, for example, the mortality risk assessed in over 900 individuals was 12.3 times higher in transgenic females compared to wild type animals (and 2.1 times higher than in tg males) [7]. This was accompanied by a slower swimming speed in males at 12 months of age, which may be comparable to the abnormal gait phenotype observed in human patients [8]. Unfortunately, the APP23-based study did not include a detailed analysis of female mice [7], so no signs of frailty were reported in the transgenic females. Conflicting data were found in the 3xTg-AD mouse model. The 3xTg-AD model is based on three mutated genes (APP Swedish, MAPT P301L, and PSEN1 M146V) and shows both amyloid and tangle pathology [9]. In this AD model, females were more severely affected by pathology, but tended to reach longevity more often than males [10]. This has been termed the morbidity/mortality paradox [11]. In an attempt to identify differences between aged transgenic mice and wild type mice and to elucidate survival parameters in AD, a small group of survivors at 18 months of age was studied (*n* = 7) [10]. Despite the marked memory deficit in the transgenic animals, no obvious differences were observed, which may be due to the rather limited group size. However, another study reported that male 3xTg-AD mice had higher frailty scores than transgenic females and also than B6129F2 wild type controls, accompanied by higher mortality [12]. Finally, in the 5xFAD mice, weight loss was described at 6 months of age compared to wild type and increased frailty scores in a female-based study [13]. Another study showed that females were less frail than males (using fixed ages of 3 and 11 months) but unfortunately did not include a comparison with wild type [14].

In recent years, research in neurodegenerative diseases has focused on the potential influence of the microbiota, mainly within the gut (reviewed in [15,16]). The gut microbiota has been shown to be associated with frailty: faecal samples from frail people had, for example, higher levels of *Akkermansia*, and lower levels of the genera *Faecalibacterium*, *Prevotella*, *Roseburia*, and *Blautia* [17]. These bacteria have also been found to be altered in AD [18]. Indeed, the transferability of AD characteristics via faeces between AD model mice and wild type mice or between affected patients and young adult rats underlines the importance of the dysbiotic gut microbiota for pathogenesis [19,20]. However, it is difficult to distinguish whether the microbiota is influenced by disease progression (chronological age) or responds to secondary effects driven by the disease (frailty). Therefore, here we aimed to analyse whether (1) frailty differs between wild type and 5xFAD mice using a natural, non-grouped colony and (2) whether chronological age or frailty affects aspects of the faecal microbiota.

Assessment of frailty in humans using multiple instruments [2] has been developed to measure the quality of ageing in clinical settings, as frail older people have higher demands on health care [3]. Early attempts in rodents included measures using specialised equipment such as the i-STAT portable clinical blood analyser [21]. Later, non-invasive parameters were defined to mimic those of clinical assessments: Whitehead and colleagues [22], for example, proposed a 31-parameter instrument to assess frailty in C57BL/6J mice (5–28 months). This instrument included evaluation of e.g., the skeletomuscular system, and also aspects of physical appearance such as coat condition. While an eight-item frailty score was not sufficient to discriminate between older adult mice and aged mice, the more detailed approach did and showed high similarity to human frailty index data. Nevertheless, such instruments need to be tailored to the respective mouse strain, as they show strain-specific differences in locomotor abilities per se and in ageing [23,24,25]. Here, we adapted frailty instruments from previous studies [14,26]) for use in 5xFAD mice of both sexes aged 3 to 16 months.

## 2. Materials and Methods

### 2.1. Animals

This study used 5xFAD transgenic mice and wild type littermates. As this model overexpresses humane genes with five mutations (the Swedish K670N, M671L, London V7171 and Florida I716V in the APP (695) gene, and M146L and L286V FAD mutations in the PS1 gene) [27], the mice are prone to develop cognitive and functional deficits as well as amyloid deposition, gliosis, and progressive neuronal loss resembling typical features of familial AD (FAD) in humans.

### 2.2. Animal Housing Conditions

Male 5xFAD mice (APP K670N, M671L, I716V; PS1 M146L, L286V; Jackson Laboratory, Bar Harbor, ME, USA) were crossbred with female C57BL/6J mice (Animal Facility of the University Medical Center Mainz) to maintain the 5xFAD and non-transgenic wild type colony (offspring were continuously crossed for more than 10 generations). Up to five mice of the same sex were housed per cage and provided with food (mouse breeding extrudate, ssniff Spezialdiäten GmbH, Soest, Germany) and water ad libitum. Mice were housed in a room with a 12 h/12 h light/dark schedule. Three-week-old offspring were ear-punched for identification and genotyping. In this study, female and male transgenic and wild type littermate mice were examined at an age between 3 to 16 months, as prior studies have shown that 5xFAD mice show early disease stages around the age of 2 to 4 months and late disease states at the age of 12 months [27]. All experimental procedures were performed in accordance with the Council Directive of the European Communities on the Care and Use of Animals for Experimental Purposes and approved by the LUA (Landesuntersuchungsamt) Rhineland-Palatinate, Germany—approval number G 19-1-025.

### 2.3. Behavioural Tests and Physical Examination

A total of 80 mice were studied, 40 of which were transgenic 5xFAD (20 female, 20 male) model mice, and 40 of which were wild type genotypes (20 female, 20 male) and therefore categorized in the control group. No fixed animal number per group was chosen but rather a cross-sectional continuous representation of different ages. Care was taken that each animal was tested only once and that animals of different ages were tested in each batch. Behavioural tests and physical examination were performed at the University Medical Center Mainz facility. The mice were divided into batches of a maximum of 11 per week and transported from the housing area to the testing room, where the tests were performed daily between 8 and 12 a.m. With the exception of the physical examination, all tests were performed in the same room with a 1-day rest period between different tests. All mice were allowed 15 min to acclimatise to the test room before the start of each procedure. The mice were provided with background music (radio) during the tests in order to prevent stress responses induced by sudden noises. Each behavioural test was repeated three times per mouse, with a minimum recovery period of 10 min between each repetition. The test arenas were disinfected with terraline solution and properly dried before the next subject was introduced. Male and female mice were tested in separate subgroups within each batch to avoid olfactory distraction. Each batch was tested according to a weekly schedule (Supplementary Table S1).

#### 2.3.1. Physical Examination and Habituation

To prevent stress induced by human influence, all mice were habituated to the investigator on the first day of the study. For this purpose, the animals were moved from the housing area to the adjacent examination room and allowed to become accustomed to the investigator’s odour and voice. Twenty parameters were used to assess physical constitution, in line with previous studies [14,26]. First, the mice were exclusively visually inspected in their home cage to assess the first 11 parameters (Supplementary Table S1). Subsequently, 9 additional parameters were assessed by lifting the mice out of the cage (e.g., visual acuity) and the mice were weighed. Each parameter was scored, with 0 points indicating a severe deficit in the parameter assessed, 0.5 points indicating a mild deficit and 1 point indicating no deficit.

Visual acuity was assessed using the visual placing response test, in which the mouse is lowered onto a plastic plate and the number of orienting trunk rotations is counted [28]. The test was repeated bilaterally (2 trials per side). Mice could score 1 point for no visual impairment (4/4 trunk rotations), or 0.5 points if the trunk was rotated 3 out of 4 times or if there was a response at least once per side. For severe impairment, 0 points were scored if the torso was not rotated at all, was rotated for only one eye, or was rotated only once in total.

Hearing function was tested by using an acoustic device that produces clicking sounds [22,29]. The device was placed at a distance of 10 cm from the subject. The clicking noise was induced after some seconds of no movement to avoid false positive results due to a visual response being mistaken for an acoustic response. The mouse’s response to the click was then tested 3 times, with a few seconds of recovery between each trial. If the mouse winced or flicked its ears in response to the sound, this was counted as a positive response. 1 point was awarded for 3/3 responses to the tone. 0.5 points were scored for 1 or 2 responses. 0 points were given for no response to the sound. To avoid prior habituation to the noise, other subjects were kept away from the test room while this test was being conducted.

#### 2.3.2. Behavioural Tests

- Open Field Test

After an acclimatisation period, mice were individually placed in the centre of a 60 × 60 × 40 cm open field arena. The total distance travelled (cm/10 min), duration of movement(s), percentage of total time spent moving, average speed (cm/s), and rearing frequency (number/min) were recorded using the ANY-maze Software 6.12 (Stoelting Europe, Dublin, Ireland) during a 10 min period [14].

- Grip-Strength Test

A Newton meter was installed to assess the grip strength of the mice, which were required to grasp a triangular bar with their forelimbs and were slowly pulled away from the apparatus until they lost grip [30]. The procedure was filmed from above with a video camera and the exact gram force (g) was recorded on the Newton meter. The test was repeated 3 times as previously described and all trial data were averaged for each individual and divided by their body weight (gram force/weight).

- Cage-Top Test

To test the motor function of the forelimbs and hindlimbs, we used the cage-top test, adapted and modified from previous publications [14,26]. Mice were suspended upside down from a cage lid. The sides were bordered with cardboard and tape to prevent the mice from climbing onto the top. The mouse´s ability to maintain grip was tested individually for 60 s. The trial was immediately repeated if a mouse fell within 10 s. The time achieved was recorded in seconds for each trial. If the mouse did not drop after 60 s, it was returned to its cage and 60 s was recorded. Each subject was tested 3 times and all trials were pooled to obtain an average. A large box of bedding was placed under the apparatus to prevent injury from falling.

- Tight-Rope Test

We used a modification of the tight-rope test [26,31], which tests motor function and coordination. Mice were placed with only their forelimbs on a 60 cm long and 1.5 cm wide cotton rope, which was installed 1 m above the ground. Due to the height, mice were unlikely to fall from the rope before showing muscular exhaustion. Subjects were monitored for a maximum of 60 s. If the mouse fell before the end of the period, the time was recorded. If the mouse managed to climb the rope and reached the end of the rope before the end of the period, a time of 120 s subtracted by the time to reach the end of the rope was recorded. If the mouse fell before reaching 10 s, the trial was immediately repeated. The average of the 3 trials was used for the final score. A large box of bedding was placed under the apparatus to prevent injury from falling.

### 2.4. Calculation of the Frailty Score

By taking the average of a physical constitution score (Pc score) based on physical examination and a musculoskeletal function score (Mf score) derived from the behavioural tests, we assigned each subject an individual frailty score.

#### 2.4.1. Physical Constitution Score

Twenty parameters were observed for each mouse and scored as described. As not every parameter was found to be commonly expressed in mice, we compared the percentage of animals affected (animals scoring 0 or 0.5 points for the parameter) for each parameter and calculated the sum of all points. The points were then averaged by dividing the sum by the number of parameters tested. To account for the varying penetrance of some parameters, the points scored for each parameter were weighed differently by multiplying them by factors corresponding to the percentage of animals affected by the parameter (Supplementary Figure S1). This allowed us to differentiate between mice affected by parameters that have been shown to be dominant in ageing mice and those that are rare. For comparison with a control group, we averaged the values for each parameter from all wild type mice with a maximum age of 6 months. We considered this group to be representative of the healthiest subgroup in our study, which should not yet show signs of ageing or frailty. Finally, the physical constitution score was calculated as follows:

$$\mathrm{Pcs}=\frac{\sum_{n=1}^{number\;of\;parameters}\left({percentual\;factor}_{n}{\times \;Score}_{parameter}\right)}{number\;of\;parameters\;observed\;\times \;average\;of\;control\;group}\times 100$$

#### 2.4.2. Musculoskeletal Function Score

The musculoskeletal function score was based on the behavioural tests performed. For the open field test, all parameters were scored individually by comparing them to the previously described control group. The grip strength, cage-top and tight-rope tests were repeated 3 times for each mouse and the average of all 3 trials was calculated and compared to the control group. Only parameters that showed a significant correlation with chronological ageing in mice were included in the final score for musculoskeletal function. The final score was calculated as follows:

$$\mathrm{Mfs}=\frac{\sum_{n=1}^{number\;of\;parameters}\left(\frac{average\;of\;3\;attempts}{average\;of\;control\;group}\times 100\right)}{number\;of\;parameters\;observed}$$

#### 2.4.3. Frailty Score

To adequately represent the development of frailty in mice (increased frailty means higher frailty score values), we used the reciprocal value of fitness. Fitness was based on the average of the musculoskeletal function score and physical constitution score. The frailty score was then calculated as follows:

$$Frailty\;score=\frac{2}{Mfs+Pcs}\times 1000\;$$

### 2.5. Microbiome Examination

#### 2.5.1. Quantification of Viable Bacteria

Representative families of gut commensals such as *Lactobacillacea*, *Enterobacteriaceae*, and Schaedler flora were analysed. One week prior to faecal collection, the mice were separated into individual cages to avoid faecal transfer.

- *Lactobacillacea* and *Enterobacteriacea*

Voluntarily provided faeces were collected from each mouse before 12 a.m. and suspended in a 0.9% sodium chloride solution at 100 μL/mg faeces [32,33]. The suspension was then homogenised using a blender (Xenox, Fähren, Germany). For *Enterobacteriaceae* and *Lactobacillaceae*, 1 mL of the diluted solution was spread on specific plates (3M Deutschland GmbH, Heidelberg, Germany) and incubated at 37 °C for 24 h. Colony forming units (CFU) were counted from whole plates for *Enterobacteriacea* or from selected representative areas for *Lactobacillaceae* and normalised to the weight of the faecal sample.

- Schaedler flora

As an anaerobic cultivatable community, bacteria grown on Schaedler agar provide an approximate overview of the major anaerobic bacterial families colonising the murine gut. We used cultivation on Schaedler agar in addition to cultivation of specific bacterial families to gain insight into the approximate amount of viable bacteria in the gastrointestinal tract. Voluntarily provided fresh faeces were suspended in 0.9% sodium chloride and mixed as described above. 10 μL of diluted solution were then plated on Schaedler agar plates (Carl Roth GmbH, Karlsruhe, Germany) and incubated anaerobically at 37 °C for 48 h (Anoxomat, Mart Microbiology B.V, Drachten, The Netherlands). Anaerobic conditions were achieved and verified by using the Anaerocult^®^ A system (VWR International GmbH, Darmstadt, Germany) and oxygen-detecting test strips (Anaerotest^®^ strips, VWR International GmbH, Darmstadt, Germany). CFU were then counted from whole plates and normalised to faecal material weight.

#### 2.5.2. qPCR of Selected Bacterial DNA

Bacterial DNA was quantified by analysis of faecal samples at the MVZ Institut fuer Mikrooekologie GmbH (Herborn, Germany). Faeces were previously collected at the University Medical Center Mainz and stored at −80 °C until samples were sent to the MVZ Institut fuer Mikrooekologie GmbH on dry ice. DNA was extracted by automated isolation using the QIAsymphony DSP Virus/Pathogen Mini-Kit on the QIAsymphony SP (QIAGEN, Hilden, Germany) and subsequently analysed [32,34]. Selected primers were used to detect whole bacterial phyla (e.g., *Firmicutes*, *Bacteroidetes*) or major representative bacterial groups (e.g., *Akkermansia muciniphila*, the genus *Bifidobacterium*). PCR was repeated three times for each sample using the ABI PRISM 7900HT sequence detection system (Applied Biosystems, Darmstadt, Germany). Each sample (25 μL) consisted of 15 ng faecal DNA, 20 pmol primer mix, and the QuantiTect SYBR Green PCR Master Mix (QIAGEN, Hilden, Germany). The detected qPCR values were quantified in bacteria per gram (wet weight) by reference to standard curves and using the average of all sequences run per sample.

### 2.6. Statistical Analysis of Data

Linear regression was fitted to the data sets to show correlation between different parameters using Pearson’s correlation coefficients (GraphPad Prism version 6 and 8, San Diego, CA, USA). Values of *p* < 0.05 were considered statistically significant. Unpaired two-sided *t*-tests were used to compare two sets of data. Outliers were identified with the ROUT method and Q = 1%.

## 3. Results

**Figure 1:**
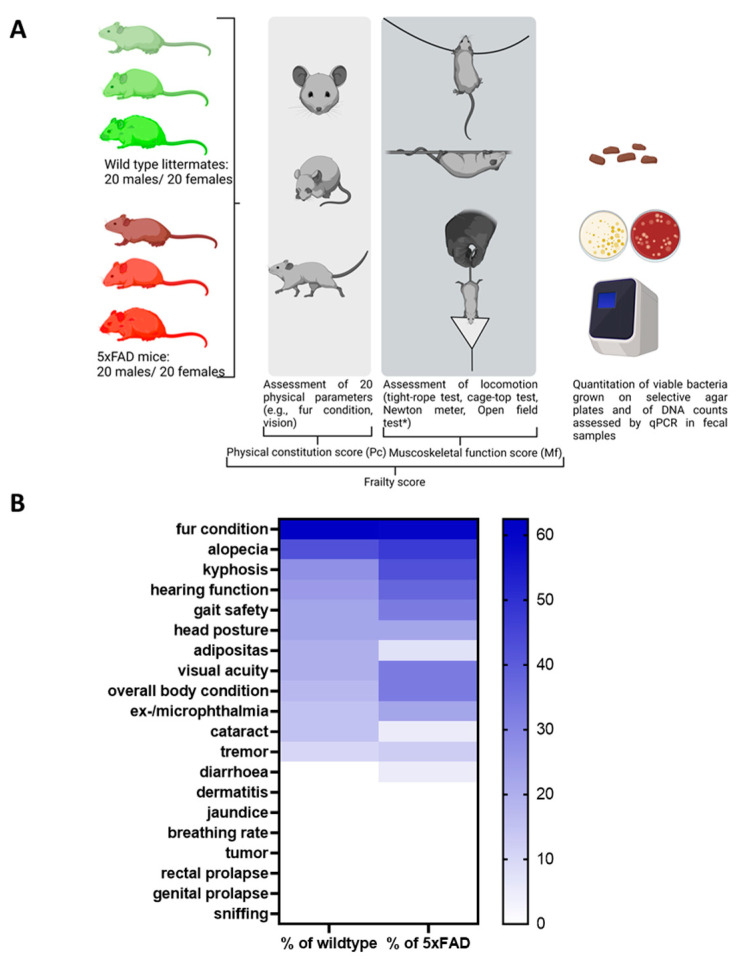
Study design and frequency of phenotypic signs of frailty in 5xFAD mice and wild type littermates. (**A**) The study was performed with 5xFAD mice and wild type littermates (*n* = 40 for both genotypes with 50% females). Animals were aged between 3 and 16 months. Rather than a fixed number of animals per group, a cross-section of different ages was selected randomly from the facility population over the course of the study. The open field test (*, not shown in the diagram) was initially considered to contribute to the Mf score. However, after evaluation in wild type mice, it was removed from the panel (see Supplementary Table S2). The figure was generated using BioRender. (**B**) Values within the heatmap are presented as percentage of occurrence in all mice of each genotype regardless of the sex or age of the animals. A colour-coded scale bar from 0 to the highest percentage obtained (62.5%) is shown on the right.

In this study, we aimed to elucidate whether chronological age or biological age (quality of ageing), i.e., frailty, is a determinant of gut microbiome composition in mice. We analysed 5xFAD Alzheimer’s disease model mice and their wild type littermates to evaluate the influence of pathological ageing processes. A cross-sectional analysis was performed using animals present in the facility at the time of study. Both sexes and animals from 3 months to 16 months of age were included (40 wild type and 40 5xFAD mice). To assess frailty, we chose to combine parameters of the physical constitution of the mice as well as sensory abilities and musculoskeletal function (Figure 1A).

### 3.1. Establishing a Frailty Score

Twenty physical parameters were tested in each subject as described in previous assessments [14,22,26], of which only 13 were found to be present in the mice studied (see Supplementary Figure S1 for an overview of all parameters tested). As shown in Figure 1B, the 5xFAD group was more severely affected than their wild type littermates for most of the parameters tested. For example, reduced hearing function was assessed in 37.5% of the 5xFAD mice, while only 25% of wild type littermates showed reduced hearing ability. Only incidences of alopecia, cataracts, and adiposity were higher in the wild type group than in the 5xFAD strain (alopecia: 42.5% and 47.5%; cataracts: 15% and 5%; adiposity: 20% and 7.5%).

**Figure 2:**
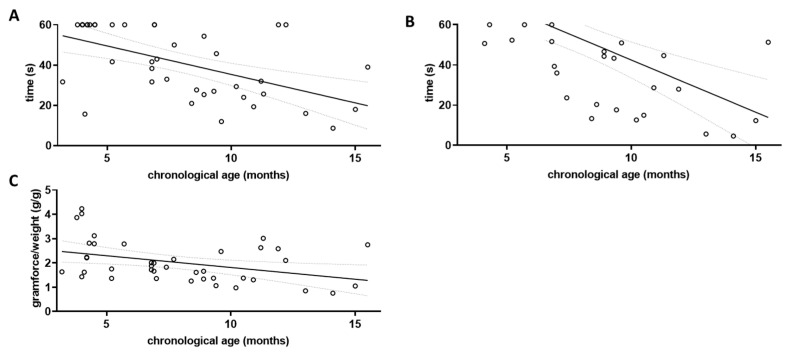
Assessment of locomotor decline in aged wild type mice. The three grip strength tests were performed on wild type littermates (wt; *n* = 40) and correlation with chronological age was investigated. Linear regression was performed and the 95% confidence interval is shown as grey dashed lines. Outlier analysis was performed using the ROUT method and Q = 1%; no outliers had to be removed (*n* = 40). (**A**) Cage-top test; (**B**) Tight-rope test; and (**C**) Grip strength measured by Newton meter.

Several tests can be used to assess locomotor function in mice, and skeletal muscle dysfunction has been described as a major cause of frailty in both human patients and mouse models [35]. To define the relevant parameters for this investigation, we first measured locomotor behaviour in four different tasks: behaviour in the open field arena, performance in the cage-top test [14,26], in the tight-rope test [26,31], and in deflecting a Newton meter. A factor indicating frailty should per se show a linear correlation with chronological age. We first tested this assumption for all four tasks using data obtained from the wild type littermates (Figure 2), as they should be indicative of normal ageing and not show accelerated ageing as might be found in the 5xFAD mice.

To assess musculoskeletal function, five qualities were tested in the open field arena, including average speed, distance travelled, rearing frequency, duration of movement, and percentage of time spent moving during a period of 10 min under camera surveillance. None of the measured parameters showed a correlation with the chronological age of the mice (see Supplementary Table S2). However, behaviour in the open field arena can also be influenced by the level of anxiety or explorative drive [36].

Grip strength was tested using three different paradigms, the cage-top test, the tight-rope test, and the grip strength assessment using a Newton meter. The results of each parameter tested were correlated with the chronological age of the wild type animals (Figure 2). For the cage-top test, a Pearson’s coefficient r of −0.5497 was obtained (*p* = 0.0002; Figure 2A), while for the tight-rope test an r of −0.6019 was obtained (*p* < 0.0001; Figure 2B). Using the Newton meter, the r for the correlation between chronological age and gram of force per gram of body weight was −0.3850 (*p* = 0.0142; Figure 2C).

**Figure 3:**
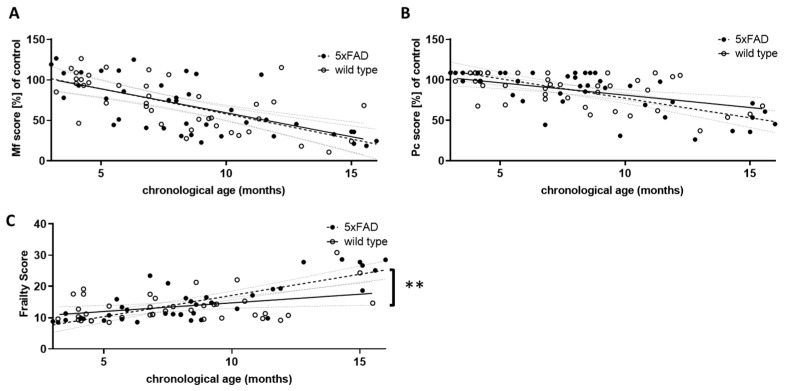
Comparison of performance scores in wild type and 5xFAD mice. The results of the three grip strength tests were used to calculate an overall musculoskeletal function score (Mf score, (**A**)), and the score points from the phenotypic signs of frailty were summed to obtain a physical condition (Pc) score (**B**). For both Mf and Pc scores, lower scores indicate poorer performance. Young wild type animals (aged 3 to 5.5 months) served as controls, and all other score values were calculated as a percentage of the control mean. Mf and Pc score were combined to give an overall frailty score (**C**). Higher scores indicate greater frailty. Outlier analysis revealed one outlier per group (Q = 1%; *n* = 39 per group). Linear regression was performed and the 95% confidence interval is indicated by grey dashed lines. Significance between slopes of the linear regression was calculated (**, *p* < 0.001).

All three grip strength tests used were found to correlate with the chronological age in wild type mice when both sexes were pooled for analysis (for separate analysis of male and female wild type mice see Supplementary Figure S2). They were therefore included in the calculation of the musculoskeletal function score for both wild type and 5xFAD mice (Mf score, Figure 3A).

Similar to the overall physical constitution score (Pc score, Figure 3B), the Mf score did not differ between 5xFAD and wild type mice with respect to the strength of correlation with chronological age. However, both were highly significantly correlated with age in individuals of both genotypes (for Mf score: r = −0.6371 (wt) and −0.6772 (5xFAD), *p* < 0.0001; for Pc score: r = −0.5169 (wt) and −0.6962 (5xFAD), *p* = 0.0006 and *p* < 0.0001). We also integrated both scores into a single frailty score (Figure 3C). This not only led to a significant correlation with chronological age for both genotypes (r = 0.3799 (wt) and 0.7880 (5xFAD), *p* = 0.0171 and *p* < 0.0001), but also to a significant difference between both genotypes, with 5xFAD showing a higher increase in frailty than wild type littermates (*p* = 0.0054). Thus, 5xFAD mice appear to age less well than their wild type littermates. Interestingly, female mice showed a milder phenotype compared to males: in wild type animals, the slope of the correlation between frailty and chronological age was significantly different (*p* = 0.0014), whereas in 5xFAD mice only a trend was observed (*p* = 0.148).

### 3.2. Analysis of the Viable Bacterial Representatives in Relation to Age and Frailty

It is known that in humans and rodents the gut microbiome is initially variable at first during childhood and puberty, then tends to be stable in both sexes if not exposed to noxae such as antibiotics, and then begins to change again with increasing age [37]. It is questionable whether chronological age or age-related functional decline are the driving forces behind the changes in older individuals. To answer this question, we first investigated two typical groups of gut bacteria by studying the families *Lactobacillaceae* and *Enterobacteriaceae*. To do this, we cultivated bacteria extracted from collected faeces on selective agar plates and counted the colony forming units (CFU). Colonising bacteria allowed us to estimate the amount of viable bacteria in the gastrointestinal tract at the time of the study. We also colonised bacteria on Schaedler agar. This allowed an estimated amount of all viable faecal bacteria to be assessed [38].

**Figure 4:**
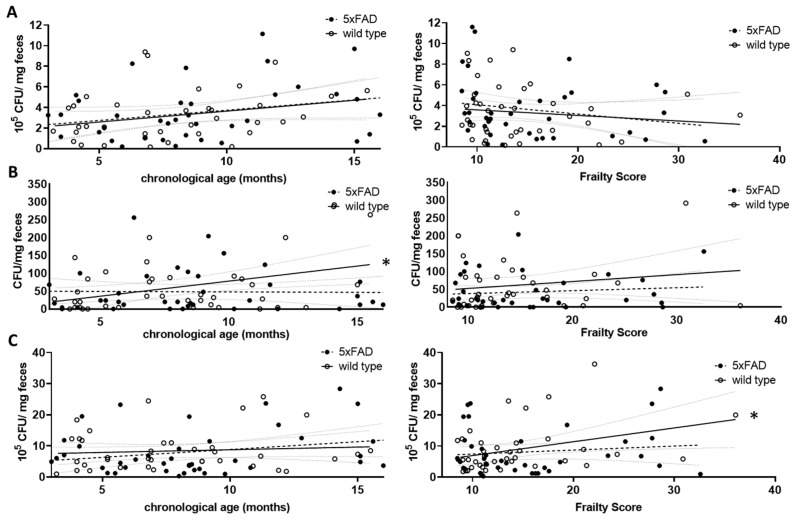
Correlation of viable bacterial subgroups with chronological age and frailty score. Voluntarily provided faecal pellets were homogenised and diluted accordingly. CFUs grown on selective agar for *Lactobacillaceae* (**A**), *Enterobacteriaceae* (**B**) or on Schaedler agar (**C**) were counted. CFU counts were analysed for correlation with chronological age (left graphs) or frailty score (right graphs). Linear regression was performed and the 95% confidence interval is indicated by grey dashed lines. Outlier analysis was performed with the ROUT method and Q = 1%. Individual data points had to be removed because they were identified as outliers or due to technical problems with plating (e.g., overgrowth of plates) (*n* = 37–40 per group). Pearson’s correlation coefficient was used for correlation analysis (*, *p* < 0.05).

For the *Lactobacillaceae* family, a non-significant increase with chronological age was found for both wild type and 5xFAD mice (*p* = 0.0895 and r = 0.2832 (wt); *p* = 0.1076 and r = 0.2688 (5xFAD); Figure 4A, left graph). This trend disappeared when the frailty score was used (*p* = 0.4334 (wt); *p* = 0.2012 (5xFAD); Figure 4A, right graph). *Enterobacteriaceae* increased significantly with age in wild type mice (r = 0.3947, *p* = 0.0129, Figure 4B, left graph), whereas no correlation between this bacterial group and age was observed in 5xFAD mice. The correlation also disappeared in wild type animals when the frailty score was taken into account (Figure 4B, right graph). Regarding the community grown on Schaedler agar, no correlation with age was observed (Figure 4C, left graph); however, a correlative association between frailty and CFU derived from cultivation on this agar was obtained in wild type animals (Figure 4C, right graph, *p* = 0.0303), whereas this did not appear in 5xFAD mice.

### 3.3. Influence of Age and Frailty on Selected Gut Microbiome Components

**Figure 5:**
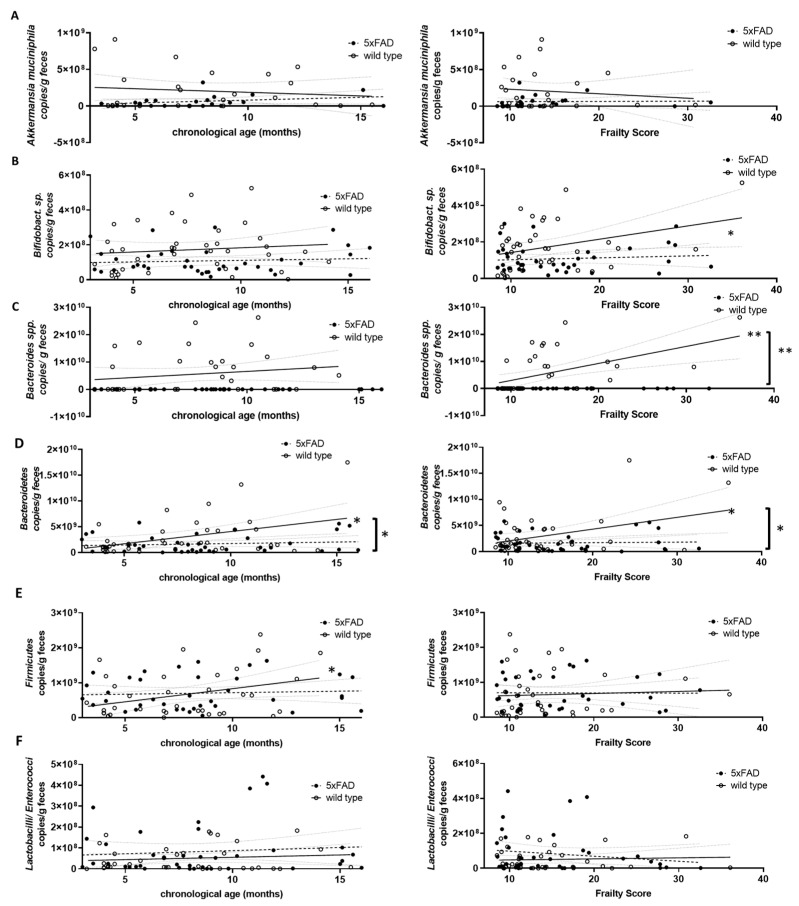
Correlation of DNA-based bacterial counts with chronological age and frailty score. Voluntarily provided faecal pellets were subjected to genomic DNA extraction and qPCR with selective primer pairs: (**A**) *Akkermansia muciniphila*; (**B**) *Bifidobacterium* sp.; (**C**) *Bacteroides* spp.; (**D**) *Bacteroidetes*; (**E**) *Firmicutes*; and (**F**) *Lactobacilli*/*Enterococci*. Counts per g faeces were analysed for correlation with chronological age (left graphs) or frailty score (right graphs). Linear regression was performed and the 95% confidence interval is indicated by grey dashed lines. Outlier analysis was performed with the ROUT method and Q = 1%. Individual data points had to be removed because they were identified as outliers or due to technical problems (below detection limit) (*n* = 21–40 per group). Pearson’s correlation coefficient was used for correlation analysis (**, *p* < 0.01; *, *p* < 0.05). Significance between slopes of the linear regression was calculated (**, *p* < 0.001; *, *p* < 0.05; indicated by brackets).

Next, we quantified several representatives of the gut microbiome using genomic DNA-based PCR and investigated a possible correlation with either chronological age or frailty. *Akkermansia muciniphila* (*Verrucomicrobia*), *Bifidobacterium* sp. (*Actinobacteria*), *Bacteroides* spp. and *Bacteroidetes*, *Firmicutes* and *Lactobacilli/Enterococci* were analysed (Figure 5). *Prevotella* (*Bacteroidetes*) and *Clostridium coccoides* (*Firmicutes*) were also analysed but resulted in a very limited number of evaluable data sets due to various samples not reaching the detection limit. Therefore, these two groups of bacteria were not considered further. Only *Firmicutes* and *Bacteroidetes*, the largest phyla in murine faecal microbiome [39], increased significantly with chronological age (Figure 5D,E, left graphs). Higher frailty scores were associated with increased *Bifidobacterium* sp., *Bacteroides* spp., and *Bacteroidetes* (Figure 5B–D, right graphs).

Differences in correlation strength for 5xFAD mice compared to wild type animals were found only for *Bacteroidetes* and age and for *Bifidobacterium* sp., *Bacteroides* spp., and *Bacteroidetes* and frailty (Figure 5D, left graph and Figure 5B–D, right graphs).

**Figure 6:**
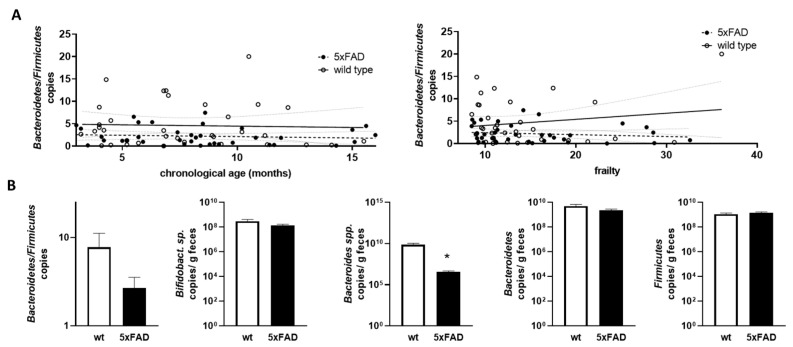
Correlation of *Bacteroidetes*/*Firmicutes* ratio with chronological age and frailty score and bacterial counts in older mice. (**A**) The Bacteroidetes/Firmicutes ratio was calculated from the data shown in Figure 5 (obtained by qPCR; D,E). The ratios were analysed for correlation with chronological age (left graph) or frailty score (right graph). Linear regression was performed and the 95% confidence interval is indicated by grey dashed lines. (**B**) Data for mice aged > 10 months were extracted and used for group-wise comparison between the 5xFAD mice and their wild type littermates (*n* = 9–12 per group). Statistical analysis was performed by using *t*-test (*, *p* < 0.05).

As the *Bacteroidetes*/*Firmicutes* ratio has been found to be informative in relation to obesity and also in comparably young 5xFAD mice [34], we next calculated this ratio based on qPCR-derived relative amounts and examined the correlation with chronological age and frailty (Figure 6A). No statistically relevant correlation or difference between the two genotypes was found.

Finally, we extracted data for mice that can be considered “old” (>10 months) [40] from datasets that contained sufficient samples (at least nine animals per group). Mice within groups had a mean age of 12.35 months to 13.32 months and did not differ significantly in age (*p* = 0.249; example for *Bacteroidetes*/*Firmicutes* ratio analysis). For the *Bacteroidetes*/*Firmicutes* ratio, there was a trend towards a lower ratio in 5xFAD mice compared to wild types, but this did not reach significance (*p* = 0.175, Figure 6B). While *Bifidobacterium* sp., *Bacteroidetes*, and *Firmicutes* were comparable between aged 5xFAD and their littermates, *Bacteroides* spp. were significantly decreased in 5xFAD mice. To summarise the data, we provide a table of significant findings (Table 1). Slightly more correlations could be found when using the frailty score and also two instead of one difference between the two genotypes could be resolved. Interestingly, the *Bacteroides*/*Bacteroidetes* groups seemed to be well represented in the results of our analysis.

## 4. Discussion

Well-ageing, also referred to as successful ageing, is now one of the main goals of preventive medicine and is in the scope of psychologists, gerontologists, and neuroscientists [41]. The chronological age alone might not be informative to report on successful ageing, as at the individual level high and stable performance in terms of memory and learning can be observed even at extreme chronological ages (e.g., centenarians [42]). One of the discussed parameters supporting or preventing successful ageing is the gut microbiome (e.g., [43]). Therefore, we analysed differences in correlations between chronological age or frailty assessed by locomotor and phenotypic testing of wild type and an AD mouse model (5xFAD) and representatives of the murine gut commensals. Our key findings were that 5xFAD mice were indeed more frail than their wild type littermates in this cross-sectional study. Interestingly, selective correlations were found when analysing either age or frailty, and only *Bacteroidetes* correlated with both in wild type mice, whereas the correlation was lost in the AD mouse model.

### 4.1. Increased Frailty in 5xFAD Mice Compared to Wild Type

Todorovic and colleagues studied frailty in 5xFAD mice without comparing them to non-transgenic mice [14]. However, they also described increased frailty in 11-month-old AD model mice as compared to 3-month-old model mice using two tools (a frailty index based on 24 parameters and a frailty score based on 8 parameters). Interestingly, while both tools described an increase in frailty in the animals, they did not congruently identify the same individuals as frail. This suggests that tools need to be carefully selected and described for such analyses. We also started our investigation by applying 20 parameters described in the literature for assessing frailty in mice, but only 13 of them were found to be relevant in the 5xFAD mouse model. Criteria for measuring frailty must therefore be adapted to each mouse strain. Todorovic and colleagues also described a milder progression of frailty in female mice compared to male mice. This was confirmed in our analysis for wild type mice and in the trend for the 5xFAD mice. The sex difference for the AD model mice did not reach statistical significance—probably because the number of female mice older than 10 months was relatively small compared to males. Nevertheless, this may reflect a general sex bias that should be taken into account when assessing frailty in mice. Surprisingly, female 5xFAD mice have been shown to have more severe pathology, as evidenced by gene expression in 4-month-old animals [44] and Aβ deposition as well as hematogenous macrophage accumulation surrounding plaques [45]. In humans, at least different sex-specific factors that maintain frailty have been identified, which could explain such differences despite a higher pathological burden in women: e.g., low weight seems to maintain frailty in men, whereas endocrine disorders seem to be more relevant in women [46]. In humans, a so-called sex–frailty paradox was described, in which women tend to live longer in general but experience higher levels of frailty than men of the same age [47]. This may be due to better coping with frailty, which ultimately results in lower vulnerability towards death. The underlying reasons for this sex-specific resilience that also seems to be present in rodent models of ageing need to be investigated in future studies.

### 4.2. Correlations of Gut Microbiota with Chronological Age or Frailty in Aged Mice

Frailty and dementia have a bidirectional relationship. Frailty phenotypes have been associated with all-cause dementia, mild cognitive impairment (MCI), AD, vascular dementia, and non-AD dementias. It appears that a higher rate of frailty is also prevalent in patients with dementia as a meta-analysis of 16 studies of older adults with AD found a prevalence of frailty ranging from 50.8% to 91.8% in acute care settings [48]. In summary, frailty is a factor that needs to be considered when studying phenotypes of older patients, but also preclinical model animals of age-related diseases such as AD. This is particularly important when the disease itself appears to contribute to frailty, as described here for AD-like pathology in the 5xFAD mouse strain.

Regarding the microbiota analysis, we found differences when assessing the correlations when only chronological age or frailty was considered. For example, cultivatable *Enterobacteriaceae* increased with chronological age in wild type mice, whereas this correlation disappeared when frailty was used as the second parameter. The increased abundance of this bacterial family confirmed findings from a previous study where aged wild type mice (1 year old) had elevated levels compared to younger mice [32]. In human studies, viable bacteria are rarely examined and results are mostly reported from sequencing. This may be due to concerns about stability and maintenance of viable conditions during storage and transportation. However, a report on 35,292 adult stool samples submitted by general practitioners for routine microbiological analysis of non-pathogen faecal bacterial flora [49], showed the same observation for humans, that *E. coli* and *Enterococcus* spp., both belonging to the *Enterobacteriaceae* group, increased with age.

Columbia blood agar was used to assess total colony forming units, which showed no changes with age. In our study in mice, total CFU measured on Schaedler agar did not increase with chronological age, but only with frailty in wild type animals. A clear limitation of our study is the comparatively small number of wild type individuals with high frailty scores that may corroborate analysis, and we cannot exclude that the lack of correlation between *Enterobacteriaceae* and frailty is due to this. However, our investigation was based on the population available within our institution at a given time and thus reflects what might be called a natural population. In very frail people, an even sevenfold increase in *Enterobacteriaceae* has been reported [50]. Therefore, we cannot exclude the possibility that a selective analysis of severely affected mice would result in a similar increase, which requires further investigation in the future.

A key finding from the PCR-based analysis was that 5xFAD mice had lower levels of *Bacteroides* spp. with respect to frailty and lower levels of *Bacteroidetes* with respect to frailty and chronological age than their wild type littermates. This is consistent with the development of a dysbiosis, as *Bacteroidetes* are considered to be one of the major representatives in the gut microbiome at all ages [51,52]. A lower abundance of this phylum compared to the abundance in healthily ageing wild type mice could indicate the growth of other non-favourable bacteria in 5xFAD mice. In elderly people from nursing homes, frailty was associated with a reduced abundance of butyrate-producing bacteria [53]. Butyrate-producers constitute >20% of the total bacterial community in adult humans and are mainly derived from *Lachnospiraceae*, *Ruminococcaceae*, and *Bacteroidetes* [54]. In addition, *Bacteroides* species such as *B. vulgatus* or *B. thetaiotaomicron* have also been associated with altered glutamate metabolism and, with this, NMDA receptor signalling [55,56]. A study with 1430 participants demonstrated a protective association of *Bacteroides* with cognitive impairment [57], which may be based on these *Bacteroides*-derived metabolites. A smaller cohort study of patients from Kazakhstan suggested even depletion of *Bacteroides* in AD [58]. However, the results are conflicting and might be due to geographic or cultural differences, at least in humans: another study described an increase of *Bacteroides* in a US AD cohort, while no effect was observed in Chinese participants [59]. Similar discrepancies have been found for mouse models of AD: in APP/PS1 mice, an increase in *Bacteroides* (more than twofold) and a therapeutic reversal by nicotinamide riboside treatment have been described [60]. A more recent study using nicotinamide treatment increased the relative amount of *Bacteroides* within the same mouse strain and interpreted this as a beneficial outcome due to the SCFA-producing abilities of these bacteria [61]. Therefore, our finding that *Bacteroides* were reduced in 5xFAD mice and uncoupled from age-dependent regulation requires further investigation. For example, a study of aged or frail 5xFAD mice under different dietary regimens would allow to confirm a general reduction of this bacterial group or to elucidate if this only occurs under certain environmental conditions (comparable to geographic localisation of humans). Moreover, a companion investigation of the metabolites would help to understand which bacteria are actively contributing to the observed state of the mouse, whether it be the pathological state or frailty status. Finally, it has to be stated that our investigation only provides correlation analyses and thus, might not address confounding factors not yet known. Another limitation of our study is that we only identified the affected bacteria as a group. *Bacteroides*, for example, include species with both beneficial and harmful effects [62]. Therefore, we cannot conclude whether they are increased as a protective strategy in ageing or as a consequence of functional decline in ageing. However, the lack of increase in 5xFAD mice indicates a disease-specific response. In this context, it is interesting to note that *B. fragilis* and its polyunsaturated fatty acid metabolites have recently been shown to activate microglia and initiate AD pathology in Thy1-C/EBPβ transgenic mice after patient-derived faecal transplantation [63]. Therefore, the observed suppression of *Bacteroides* in 5xFAD mice might be a compensatory mechanism to control or combat pathology. However, this needs to be investigated by using more fine-grained methods to allow for species and even strain subtype discrimination and assessing activity of found bacteria in the ecological niche of the gut.

## 5. Conclusions

Cross-sectional analysis of the murine faecal microbiota revealed that not only chronological ageing but also quality of ageing can possibly drive the composition of gut commensals. Changes that occur during normal ageing (wild type) can be overwhelmed by a pathological ageing phenotype with increased frailty, as shown here for the AD mouse model. *Bacteroides* were identified as a highly informative bacterial candidate group that increased in non-pathological ageing (wild type) with frailty.

## Figures and Tables

**Table 1:** Statistically significant correlations found for bacterial groups and either chronological ageing or frailty score. The direction of the correlation is indicated by the direction of the arrowhead. Correlations where the linear regression slopes significantly differed are indicated by a ‘+’.

Bacterial Subgroup	Wild Type		5xFAD		Difference between 5xFAD and Wild Type	
	Chron. Age	Frailty Score	Chron. Age	Frailty Score	Chron. Age	Frailty Score
*Enterobacteriaceae* (CFU)	↑	-	-	-	-	-
Schaedler (CFU)	-	↑	-	-	-	-
*Bifidobacterium* sp.	-	↑	-	-	-	-
*Bacteroides* spp.	-	↑	-	-	-	+
*Bacteroidetes*	↑	↑	-	-	+	+
*Firmicutes*	↑	-	-	-	-	-

## Data Availability

Data are contained within the article or Supplementary Materials.

## Supplementary Materials

The following supporting information can be downloaded at: https://www.mdpi.com/article/10.3390/microorganisms11122856/s1, Figure S1: Tested parameters (derived from [14,26]) with percentage of occurrence in all examined mice; Figure S2: Assessment of locomotive functional decline in aged male and female wild type mice; Table S1: Schedule of examination of mice; Table S2: Parameters measured in the open field arena in wild type littermates.
