# Supplementary material for: Comparison of Frailty and Chronological Age as Determinants of the Murine Gut Microbiota in an Alzheimer’s Disease Mouse Model
Source: Microorganisms. 2023 Nov 24;11(12):2856. doi: 10.3390/microorganisms11122856 (PMC10745811; doi:10.3390/microorganisms11122856)
Supplement: Supplementary file 1 [file microorganisms-11-02856-s001.zip › Suppl. Figure S1.pdf]

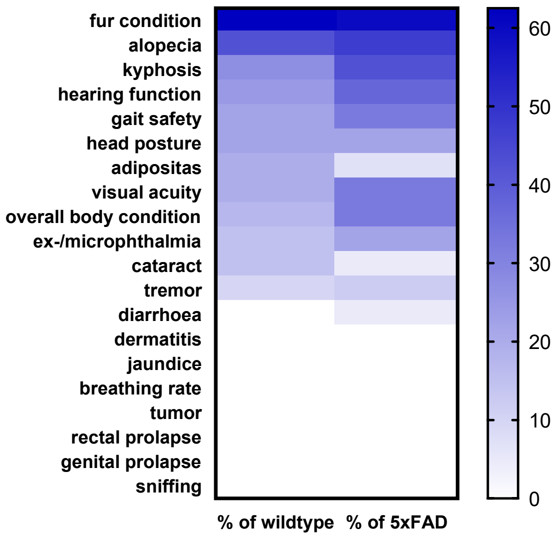

Suppl. Fig. S1: Tested parameters (derived from Todorovic et al., 2020 and Kane et al., 2016) with percentage of occurrence in all examined mice. All parameters that were never observed in both genotypes (wild type and 5xFAD) were excluded from calculating the frailty score.
