# Supplementary material for: Comparison of Frailty and Chronological Age as Determinants of the Murine Gut Microbiota in an Alzheimer’s Disease Mouse Model
Source: Microorganisms. 2023 Nov 24;11(12):2856. doi: 10.3390/microorganisms11122856 (PMC10745811; doi:10.3390/microorganisms11122856)
Supplement: Supplementary file 1 [file microorganisms-11-02856-s001.zip › Suppl. Figure S2.pdf]

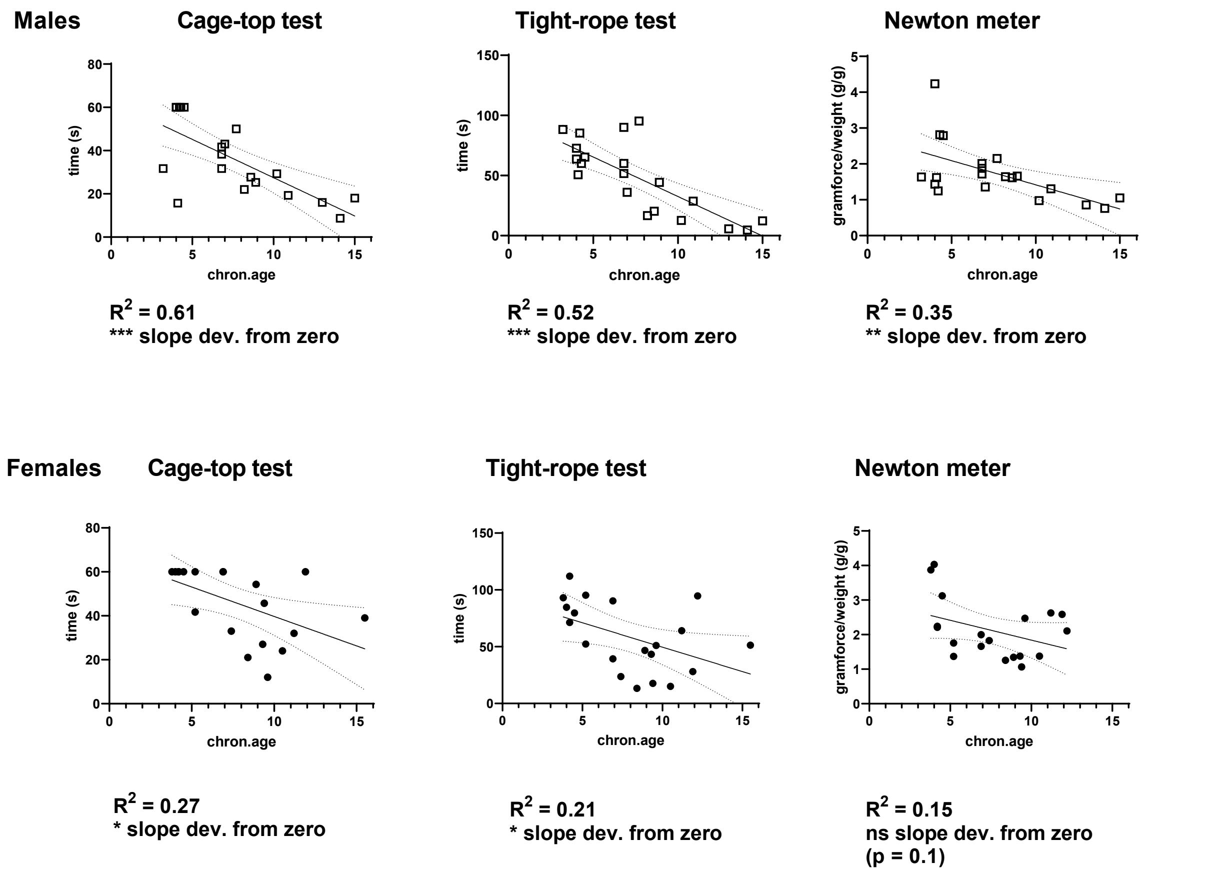

Suppl. Fig. S2: Assessment of locomotive functional decline in aged male and female wild type mice. The three grip strength tests were applied on wild type littermates and correlation with chronological age investigated. Linear regression was performed and 95% confidence interval is indicated by dashed lines. Outlier analysis was performed with ROUT method and Q = 1%; one outlier had to be removed from female values for cage-top test and Newtonmeter testing (n=19-20 per group).
