# Supplementary material for: Comparison of Frailty and Chronological Age as Determinants of the Murine Gut Microbiota in an Alzheimer’s Disease Mouse Model
Source: Microorganisms. 2023 Nov 24;11(12):2856. doi: 10.3390/microorganisms11122856 (PMC10745811; doi:10.3390/microorganisms11122856)
Supplement: Supplementary file 1 [file microorganisms-11-02856-s001.zip › Suppl. Table S1.pdf]

**Parameter**	**Assessed**
Alopecia (back)	In home cage
Dermatitis (back)	In home cage
Fur condition (back)	In home cage
Gait safety	In home cage
Tremor	In home cage
Hearing function	In home cage
Head posture	In home cage
Microphthalmia/ Exophthalmia	In home cage
Cataract	In home cage
Jaundice	In home cage
Breathing rate/ depth	In home cage
Tumor (back)	In home cage when rearing
Overall body condition	In home cage when rearing
Kyphosis	In home cage when rearing
Adiposity (size of abdomen)	In home cage when rearing
Rectal prolapse	Removal from cage
Diarrhea	Removal from cage
Alopecia (ventral)	Removal from cage
Dermatitis (ventral)	Removal from cage
Fur condition (ventral)	Removal from cage
Tumor (ventral)	Removal from cage

Genital prolaps	Removal from cage
Sniffing	Removal from cage
Visual acuity	Removal from cage

Suppl. Table S1: Schedule of examination of mice.

Parameters that were singly observed for back – and ventral side of the mouse were combined in the heatmaps as e.g., fur condition in general.
