# Supplementary material for: Comparison of Frailty and Chronological Age as Determinants of the Murine Gut Microbiota in an Alzheimer’s Disease Mouse Model
Source: Microorganisms. 2023 Nov 24;11(12):2856. doi: 10.3390/microorganisms11122856 (PMC10745811; doi:10.3390/microorganisms11122856)
Supplement: Supplementary file 1 [file microorganisms-11-02856-s001.zip › Suppl. Table S2.pdf]

**Parameter**	**Pearson r**	**P value**
average velocity	-0.004087	0.9800
duration of movement	-0.07067	0.6648
traveled distance	-0.008743	0.9573
rearing frequency	-0.08106	0.6190
percentage of time spent moving during a period of 10 minutes	-0.2432	0.1305

Suppl. Table S2: Parameters measured in the open field arena in wild type littermates. Data of 40 mice were used for correlation analysis with chronological age. None of the parameters achieved statistical significance.
